# Hyperpolarized Magnetic Resonance and Artificial Intelligence: Frontiers of Imaging in Pancreatic Cancer

**DOI:** 10.2196/26601

**Published:** 2021-06-17

**Authors:** José S Enriquez, Yan Chu, Shivanand Pudakalakatti, Kang Lin Hsieh, Duncan Salmon, Prasanta Dutta, Niki Zacharias Millward, Eugene Lurie, Steven Millward, Florencia McAllister, Anirban Maitra, Subrata Sen, Ann Killary, Jian Zhang, Xiaoqian Jiang, Pratip K Bhattacharya, Shayan Shams

**Affiliations:** 1 Department of Cancer Systems Imaging University of Texas MD Anderson Cancer Center Houston, TX United States; 2 Graduate School of Biomedical Sciences University of Texas MD Anderson Cancer Center Houston, TX United States; 3 School of Biomedical Informatics University of Texas Health Science Center at Houston Houston, TX United States; 4 Department of Electrical and Computer Engineering Rice University Houston, TX United States; 5 Department of Urology University of Texas MD Anderson Cancer Center Houston, TX United States; 6 Department of Translational Molecular Pathology University of Texas MD Anderson Cancer Center Houston, TX United States; 7 Department of Clinical Cancer Prevention University of Texas MD Anderson Cancer Center Houston, TX United States; 8 Department of Pathology University of Texas MD Anderson Cancer Center Houston, TX United States; 9 Division of Computer Science and Engineering Louisiana State University Baton Rouge, LA United States

**Keywords:** artificial intelligence, deep learning, hyperpolarization, metabolic imaging, MRI, 13C, HP-MR, pancreatic ductal adenocarcinoma, pancreatic cancer, early detection, assessment of treatment response, probes, cancer, marker, imaging, treatment, review, detection, efficacy

## Abstract

**Background:**

There is an unmet need for noninvasive imaging markers that can help identify the aggressive subtype(s) of pancreatic ductal adenocarcinoma (PDAC) at diagnosis and at an earlier time point, and evaluate the efficacy of therapy prior to tumor reduction. In the past few years, there have been two major developments with potential for a significant impact in establishing imaging biomarkers for PDAC and pancreatic cancer premalignancy: (1) hyperpolarized metabolic (HP)-magnetic resonance (MR), which increases the sensitivity of conventional MR by over 10,000-fold, enabling real-time metabolic measurements; and (2) applications of artificial intelligence (AI).

**Objective:**

Our objective of this review was to discuss these two exciting but independent developments (HP-MR and AI) in the realm of PDAC imaging and detection from the available literature to date.

**Methods:**

A systematic review following the PRISMA extension for Scoping Reviews (PRISMA-ScR) guidelines was performed. Studies addressing the utilization of HP-MR and/or AI for early detection, assessment of aggressiveness, and interrogating the early efficacy of therapy in patients with PDAC cited in recent clinical guidelines were extracted from the PubMed and Google Scholar databases. The studies were reviewed following predefined exclusion and inclusion criteria, and grouped based on the utilization of HP-MR and/or AI in PDAC diagnosis.

**Results:**

Part of the goal of this review was to highlight the knowledge gap of early detection in pancreatic cancer by any imaging modality, and to emphasize how AI and HP-MR can address this critical gap. We reviewed every paper published on HP-MR applications in PDAC, including six preclinical studies and one clinical trial. We also reviewed several HP-MR–related articles describing new probes with many functional applications in PDAC. On the AI side, we reviewed all existing papers that met our inclusion criteria on AI applications for evaluating computed tomography (CT) and MR images in PDAC. With the emergence of AI and its unique capability to learn across multimodal data, along with sensitive metabolic imaging using HP-MR, this knowledge gap in PDAC can be adequately addressed. CT is an accessible and widespread imaging modality worldwide as it is affordable; because of this reason alone, most of the data discussed are based on CT imaging datasets. Although there were relatively few MR-related papers included in this review, we believe that with rapid adoption of MR imaging and HP-MR, more clinical data on pancreatic cancer imaging will be available in the near future.

**Conclusions:**

Integration of AI, HP-MR, and multimodal imaging information in pancreatic cancer may lead to the development of real-time biomarkers of early detection, assessing aggressiveness, and interrogating early efficacy of therapy in PDAC.

## Introduction

There is an unmet need for noninvasive surrogate markers that can help to identify the aggressive subtype(s) in a pancreatic lesion at an early time point [1]. In contrast to the declines in cancer-related deaths from other malignancies, progress in the management of pancreatic ductal adenocarcinoma (PDAC) has been slow, and the incidence of cancer-related deaths due to PDAC continues to rise [2]. PDAC develops relatively symptom-free, and is one of the leading causes of cancer-related deaths in the United States. In 2020 alone, it was estimated that approximately 57,600 people (30,400 men and 27,200 women) would be diagnosed with PDAC, and approximately 47,050 people (24,640 men and 22,410 women) were projected to die of the disease [3]. Early detection of PDAC is unusual and typically incidental, with the majority (~85%) presenting with locally advanced or metastatic disease when surgery, the only curative modality, is not an option. Overall, PDAC is associated with a dire prognosis and a 5-year survival rate of only 8% [3]. The absence of early symptoms and lack of a reliable screening test have created a critical need for identifying and developing new noninvasive biomarkers for the early detection of PDAC [1].

Hyperpolarization (HP)-based magnetic resonance (MR) has become a major new imaging modality by providing valuable information on previously inaccessible aspects of biological processes owing to its ability for detecting endogenous, nontoxic ^13^C-labeled probes that can monitor enzymatic conversions through key biochemical pathways [4-6]. Clinical trials with this modality are ongoing at several centers worldwide [7]. HP-MR provides an exciting opportunity to identify and understand early metabolic aberrations, enabling the detection of advanced pancreatic preneoplastic lesions and PDAC at the smallest size for which no methods of detection currently exist. In general, cancer, and PDAC in particular, is considered a paradigm of genetically defined metabolic abnormalities. Genetic mutations can trigger specific signaling pathways that are associated with metabolic transformations, which can potentially be detected by HP methods with a high degree of sensitivity.

In conventional MR, the signal measured is generated from the abundance of hydrogen in the body, specifically water [8]. Organic molecules at high concentration in the body with a high abundance of hydrogens such as choline, lipids, and lactate can also be measured using MR. Other nuclei such ^13^C and ^15^N can also be measured using MR, but their utility in living systems is low due to their low abundance in nature (the natural abundance of ^13^C is 1%) and their smaller gyromagnetic ratio compared to that of hydrogen [9]. HP enables these nuclei to be observed in vivo.

HP allows for >10,000-fold sensitivity enhancement relative to conventional MR, and is a nontoxic, nonradioactive method for assessing tissue metabolism and other physiological properties [10-13]. There are four established methods for producing HP probes: (i) dynamic nuclear polarization (DNP) [10,13], (ii) optical pumping of noble gases [14], (iii) the brute force approach [15], and (iv) parahydrogen-induced polarization [16]. The detailed physics of these HP methods can be found elsewhere [17]. The most common and widely used method for HP is DNP, in which magnetization is transferred from the unpaired electrons (usually from added radicals) to the isotopically labeled probe [17]. This transfer of magnetization occurs under microwave irradiation at a low temperature of 1.5 K and a high magnetic field of 3 T. Development of the dissolution DNP technique in 2003 [4] opened a new avenue to monitor in vivo metabolism, enabling the detection and tracking of the fate of metabolites containing low-abundance nuclei such as ^13^C [18]. The routine dissolution DNP instrument employed, which carries out HP in the preclinical setting, is HyperSense (Oxford Instruments, UK), as shown in Figure S1 in Multimedia Appendix 1. A clinical polarizer is available for performing real-time metabolic profiling in humans (SPINLab, GE Healthcare) and over 20 such polarizers have been installed worldwide [19].

The most commonly used HP probes to track the pathways of interest are ^13^C-enriched probes, which are either uniformly or selectively enriched. The other reason to employ ^13^C-enriched molecules is the comparatively longer longitudinal relaxation time (T_1_) of the ^13^C nucleus compared to that of other nuclei. The high ^13^C signal of HP probes and the fact that an HP signal is carried over in the products of biochemical transformation allow investigators to interrogate biochemical reactions in real time. These probes are usually part of essential biochemical reactions such as glycolysis (glucose and pyruvate) and the tricarboxylic acid (TCA) cycle (succinate, fumarate, and glutamine).

HP-MR experiments have been performed mostly in preclinical models to date, and HP-MR is not currently routinely used in clinical settings. However, several clinical trials have been performed or are ongoing [5]. HP-MR in the preclinical setting involves injecting the HP probe dissolved in a biocompatible solvent into the tail vein of rodents. The probe diffuses through the blood to populate in well-perfused body tissues. After entering the extracellular fluid, the molecule is taken up into the cells with the help of endogenous transporters. All of these processes must occur before the HP signal decays, which is determined by the decay time (ie, T_1_) of the HP probe. For most probes, T_1_ ranges from 15-20 seconds to approximately 1 minute. Hence, it is important to dissolve the probe in the solvent immediately and inject into the animals quickly to avoid loss of the HP signal due to relaxation. A specially designed proton volume coil and ^13^C surface coil are used to receive the signal from the enriched HP ^13^C probe in vivo.

The utility of HP-MR is not only simply tracking the probe diffusing inside the body but also its ability to visualize downstream metabolic products of injected probes converted by endogenous enzymes [5]. HP-MR can be used to quantify in vivo metabolic flux in real time. However, all processes must be completed within the time frame of T_1_ of the HP probe. Therefore, only relatively fast biochemical reactions can be visualized.

**Figure 1:**
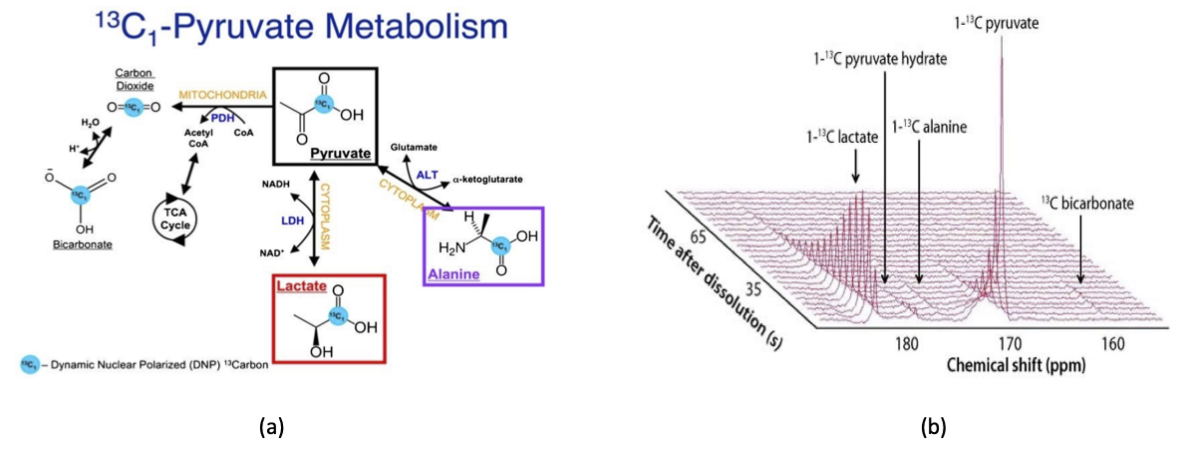
(a) Schematic showing pyruvate metabolism inside a cell. The [1-13C] pyruvate can be converted to 13C-lactate, 13C-alanine, and 13C-bicarbonate in the presence of enzymes lactate dehydrogenase-A (LDHA), alanine transferase (ALT), and pyruvate decarboxylase, respectively. (b) Downstream products of pyruvate metabolism such as lactate and alanine can be imaged using hyperpolarized magnetic resonance. A 3D, real-time readout of the signals, as shown here, can be created using standard software such as Chenomx.

Glycolysis (the breakdown of glucose) is a multistep process that eventually yields pyruvate in the cytosol. Pyruvate is the final breakdown product of glucose in glycolysis and is preferably converted to lactate. The high dependence of cancer cells on glucose and glycolysis is often referred to as the Warburg effect after the initial discovery of this dependence by Dr. Otto Warburg [20]. Therefore, HP [1-^13^C]-pyruvate is the most common HP probe for determining glycolytic flux in cancer. Another key point is that pyruvate is taken up rapidly by monocarboxylate transporters [21]. In the cytosol, the HP pyruvate has four important fates [22]: (i) conversion to lactate; (ii) conversion to alanine; (iii) transport into the mitochondria and conversion to carbon dioxide; and (iv) conversion to acetyl-coenzyme A to be utilized in the TCA cycle, which can be tracked by labeling the first carbon of pyruvate (Figure 1a). When HP-pyruvate is injected into an animal, the signal is recorded from an anatomical imaging slice placed in the tissue of interest. An example of a metabolic HP-MR spectrum is shown in Figure 1b. The flux from pyruvate to a downstream metabolite can be visualized and evaluated using either TopSpin (Bruker BioSpin GmbH) or MestReNova (Mestrelab Research) in either of the two following ways: by measuring the ratio of signals integrated over time (eg, lactate-to-pyruvate ratio, alanine-to-lactate ratio) [23] or by calculating the Kp value (according to the Bolch equation): K_PL_ (pyruvate to lactate) and K_PA_ (pyruvate to alanine) [24].

In summary, HP-MR provides a unique opportunity to measure real-time metabolic signals arising in the tissue of interest with over 10,000-fold sensitivity enhancement that cannot be interrogated by other imaging techniques. The provided outcome is the spectroscopic signatures of the metabolites of interest that are recorded as resonances at different and unique chemical shifts (Figure 1b). The HP-pyruvate signal undergoes decay once it is hyperpolarized with a characteristic decay constant (T_1_ ~50 seconds) as well as the downstream products of the metabolism (eg, alanine and lactate). Overall, there is a time window of 3×T_1_ (~150 seconds) to accomplish this real-time metabolic imaging, and this short time frame is a major limitation of HP-MR. Fast MR sequence design along with powerful and rapid imaging gradients can help in acquiring more sensitive and informative spectra in the future to mitigate this limitation. Several MR imaging (MRI) companies such as GE Healthcare, Siemens, and Bruker have devoted considerable research investment on this matter.

Artificial intelligence (AI) is a fast-developing research field in which machines are utilized to learn from observations to mimic human intelligence. Kaplan et al [25] define AI as a system’s ability to correctly interpret external data, to learn from such data, and to use those learnings to achieve specific goals and tasks through flexible adaptation. Over the last decade, deep learning has dramatically reshaped AI research. With the development of deep learning, a subfield of AI, and recognition of its potential in feature extraction and flexibility, it has increasingly been applied to numerous medical scenarios such as diagnosis, health care delivery optimization, genomics, and drug discovery [26-31]. Machine learning has been utilized for online health care management [32], disease prevention [33], clinical note processing [34], and management of chronic diseases [35]. AI has been leveraged for diagnosis and localization of regions of interest (ROIs) using a vast array of medical images such as optical images, MRI, X-rays, and computed tomography (CT) [36-41]. As a result, there is a great opportunity to utilize AI for the early detection of cancer such as PDAC.

Deep-learning algorithms rely on neural networks, which mimic the process of information transformation by neurons in the biological brain [42]. Neural networks adaptively learn features from observations during training and translate the input data to high-dimensional representations suitable for classification or regression tasks. The success of deep-learning algorithms is rooted in their multiple stacked layers and efficient feature extraction, often explained as a powerful representation learning method. Each layer consists of multiple neurons transforming the information nonlinearly by an activation function. This architecture allows for high-level interactions between transformed features coming from the previous layers to contribute to the output. Hence, deep-learning algorithms could automatically optimize the parameters and learn a high-level representation of input data aligned with the target task.

**Figure 2:**
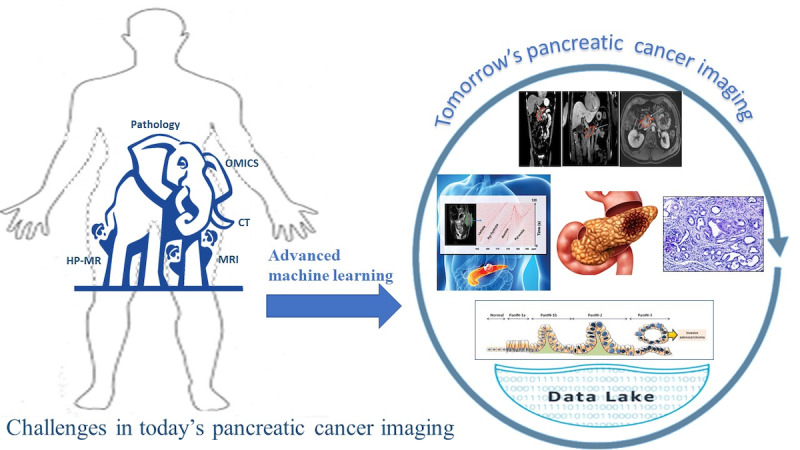
Cartoon showing the challenges of imaging pancreatic cancer at early stages and how artificial intelligence can interface with hyperpolarized magnetic resonance (HP-MR), anatomical magnetic resonance imaging (MRI), and pathology data toward developing biomarkers of pancreatic cancer premalignancy. This approach may become the standard of care in the clinic of the future. CT: computed tomography.

As shown in Figure 2, we believe that the knowledge gap of “early diagnosis of pancreatic cancer with noninvasive imaging” is an elephant in the dark that cannot be accomplished with a single modality. Pancreatic cancer at the very early stages is completely asymptomatic. Conventional anatomical imaging cannot detect any of these early stages of premalignancy of this deadly disease when therapeutic or early surgical interventions can be most effective. Conventional MRI can detect intraductal papillary mucinous neoplasms (IPMNs) where epithelial pancreatic cystic tumors of mucin-producing cells arise from the pancreatic ducts [43]. Although IPMNs are benign tumors, they can progress to pancreatic cancer in some cases [43]. However, MRI as well other imaging modalities fail to detect any other premalignant lesions such as pancreatic intraepithelial neoplasia (PanIN), which is a more commonly accepted mechanistic pathway of the tumorigenesis of PDAC [44]. It is important to recognize that an individual with even stage I (localized) pancreatic cancer has a 5-year survival rate of only 39% [45]. This emphasizes the point that early detection in pancreatic cancer must occur at stages earlier than clinical stage I.

HP-MR can detect metabolic changes at very early stages of lesion formation in the pancreas; however, this is more of an MR spectroscopic technique than an MRI modality. Moreover, the signal from HP compounds lasts no more than a few minutes that allow for a rapid acquisition of dynamic metabolic flux measurements in the organ of interest. This review will focus on the introduction of AI approaches to CT and MRI datasets, and the applications of HP-MR in pancreatic cancer. In the Results section, we summarize the strengths and weaknesses of each technique, and discuss our solution to leverage the unique strengths of AI to learn biomarkers from both HP-MR and MRI modalities, in addition to the available pathology and immunohistochemistry data to bridge this crucial knowledge gap. Our laboratories are currently pursuing an AI approach using an HP-MR dataset as applied to PDAC, the results of which will be published in the near future. In addition, we discuss the broad range of HP probes used to interrogate physiological functions such as metabolism and pH, which may expand the scope of applying AI to the functional imaging of PDAC.

## Methods

A systematic review was performed following the PRISMA (Preferred Reporting Items for Systematic Reviews and Meta-Analyses) extension for scoping reviews (PRISMA-ScR) guidelines. Studies addressing the utilization of HP-MR and/or AI for early detection, assessment of aggressiveness, and interrogation of the early efficacy of therapy in patients with PDAC cited in recent clinical guidelines were extracted from the PubMed and Google Scholar databases. The studies were reviewed following predefined exclusion and inclusion criteria, which were grouped based on the utilization of HP-MR and AI in PDAC diagnosis.

Application of the HP-MR technique in pancreatic cancer is still nascent. We have reviewed every paper published in this broad area up to November 2020. Taken together, we have summarized our review in two tables. Table 1 summarizes all ^13^C-labeled HP probes employed in interrogating different metabolic pathways in pancreatic cancer systems, and Table 2 summarizes all published applications of HP-MR in preclinical models of PDAC. In all, we have classified all of the physiological applications of HP-MR in pancreatic cancer under seven categories. The details of the deep-learning methods and HP-MR in different PDAC applications are discussed in the Introduction section above and in the relevant subsections of the Results.

**Table 1:** Review of 13C-labeled probes employed in interrogating different metabolic pathways in pancreatic cancer systems.

HP^a^ probe	Biochemical reaction	T_1_^b^ of HP probe (seconds)	Quantification	Biological significance	References
[1-^13^C] Pyruvate	Pyruvate to lactate (catalyzed by LDH^c^); pyruvate to alanine (catalyzed by ALT^d^)	44-67	Rate constant of pyruvate to lactate (or alanine) or time-integrated ratio of lactate (or alanine)-to-pyruvate signals	Increased pyruvate-to-lactate flux is an indicator of the Warburg effect; total flux from pyruvate to (lactate+alanine) could be a measure of anaerobic glucose metabolism	Viale et al [17], Rao et al [22], Halbrook and Lyssiotis [49], Dutta et al [50]
[5-^13^C] or [5-^13^C-4-^2^H2] glutamine	Glutamine to glutamate (catalyzed by glutaminase)	16-30	Time-integrated ratio of glutamate-to-glutamine signals	Indicator of glutamine addiction as a characteristic of certain cancers; also a measure of α-ketoglutarate metabolism (glutamate converts to α- ketoglutarate and can feed the TCA^e^ cycle).	Son et al [51]
[H^13^CO_3_^–^] bicarbonate	Bicarbonate to carbon dioxide	10-20	Using the relative concentrations of bicarbonate and carbon dioxide, apply the Henderson-Hasselbalch equation to calculate the tissue pH	The bicarbonate buffer system controls tissue pH; greater acidity of the tumor microenvironment has been linked to treatment resistance	Cruz-Monserrate et al [52], Gallagher et al [53]
[1,5-^13^C_2_] zymonic acid	N/A^f^	43-51	Chemical shift difference based on pH measurement	This is an organic moiety with no significant biological importance	Rao et al [21]
[1,4-^13^C_2_] fumarate	Fumarate to malate (cytosolic washout after cell necrosis)	~30	Malate signal is proportional to the amount of cell death	Fumarase (FH) enzyme is present in the cytosol and mitochondria of viable cells. Since cells cannot uptake fumarate, any HP malate production is a direct result of injected HP fumarate interacting with FH in the extracellular space, which has leaked out of necrotic cells; thus, it can be used to differentiate necrotic from viable cells	Silvers et al [54], Lee et al [55]
[1-^13^C] dehydroascorbate (DHA)	DHA/ascorbate cycle, GSH^g^/GSSG^h^ cycle, and NAPDH^i^ to NADP+	>50	Ratio of time-integrated ascorbate-to-DHA signal	Greater flux from DHA to ascorbate indicates less redox stress inside the cell; this is also an indirect measure of the GSSG-to-GSH ratio and NADPH metabolism	Lai et al [56], Salamanca-Cardona et al [57], Keshari et al [58-60]
[1-^13^C] α-keto isocaproate (α-KIC)	α-KIC to leucine (catalyzed by BCAT^j^)	100	Ratio of time-integrated leucine-to-α-KIC signals	Indicator of BCAT level, which is upregulated in certain cancers	Wilson et al [61]

^a^HP: hyperpolarization.

^b^T_1_: longitudinal relaxation time.

^c^LDH: lactate dehydrogenase.

^d^ALT: alanine transaminase.

^e^TCA: tricarboxylic acid cycle.

^f^N/A: not applicable.

^g^GSH: reduced glutathione.

^h^GSSG: glutathione disulfide.

^i^NAPDH: nicotinamide adenine dinucleotide phosphate.

^J^BCAT: branched-chain aminotransferase.

**Table 2:** Review of published applications of hyperpolarized magnetic resonance (HP-MR) in preclinical pancreatic ductal adenocarcinoma (PDAC) models.

Purpose of study	Mouse model/cell line/site of injection	HP-MR probe and downstream reaction	Results	Implications for HP-MR	Reference
To investigate whether pancreatic preneoplasia can be detected prior to the development of invasive cancers in GEM^a^ models of PDAC using HP-MR.	I. For early-onset PDAC: GEM (K-Ras and p53 mutations); cell line II. For late-onset PDAC: GEM (only K-Ras mutation); cell line III. Wild-type mice; pancreatitis induced using caerulein injection	[1-^13^C] pyruvate Pyruvate to lactate and pyruvate to alanine	I. The alanine-to-lactate signal ratio decreases progressively from the normal pancreas to pancreatitis to low-grade PanIN^b^ to high-grade PanIN to PDAC, using HP-MR II. Holds true for individual mice with time as well as upon comparing the three groups, considering their genetic proximity to PDAC (I>II>III) III. Caused by increasing LDH^c^ activity and decreasing ALT^d^ activity	Clinical potential for early detection of advanced pancreatic preneoplasia in high-risk patients using the alanine-to-lactate signal ratio as a biomarker. Diseased areas can be monitored over time. Kinetic rate constants (k_PA_ and k_PL_) can be used as metabolic imaging biomarkers of pancreatic premalignant lesions	Düwel et al [22], Dutta et al [50]
I. To determine if HP-MR can inform the sensitivity of pancreatic tumors to the hypoxia-activated prodrug TH-302 II. To test whether an adjuvant injection of pyruvate would enhance TH-302 efficacy	I. In female SCID mice: (i) highly sensitive to TH-302: SC^e^ injection of the PDX^f^ Hs766t; (ii) moderately sensitive to TH-302: SC injection of the PDX MIAPaCa-2; (iii) resistant to TH-302: SC injection of the PDX SU.86.86 II. Treatment groups: (i) Control, (ii) TH-302, (iii) TH-302+pyruvate	[1-^13^C] Pyruvate Pyruvate to lactate	I. Higher lactate-to-pyruvate ratio observed in Hs766t and MIAPaCa groups; lower lactate-to-pyruvate ratio in SU.86.86 group II. Treatment with only TH-302: response of Hs766t (highly sensitive)> MIAPaCa-2> SU.86.86 (resistant). Treatment with TH-302+pyruvate: Hs766t and MIAPaCa-2 respond to a greater extent; SU.86.86 still resistant III. Exogenous pyruvate would be a successful adjuvant to enhance TH-302 efficacy because it stimulates oxygen consumption in glycolytic cells and decreases tumor pO_2_ transiently	HP-MR can be used to predict treatment response to hypoxia-activated prodrugs, and thus provide a prognostic biomarker	Stødkilde-Jørgensen et al [63]
I. To determine a genetic biomarker of the response to the LDH-A inhibitor FX11 II. To test the response of HP-MR output to FX11 in PDAC murine models	I. In male nu/nu athymic mice: SC injection of PDX of PDAC with (i) wild-type TP53 or (ii) mutant TP53 II. Treatment groups: (i) Control, (ii) FX11	[1-^13^C] Pyruvate Pyruvate to lactate	I. Mice injected with mutant TP53 PDAC responded to FX11; those injected with wild-type TP53 did not respond to FX11 by the end of 4 weeks II. The TP53 target gene *TIGAR* was responsible for the lack of response in wild-type TP53 PDAC. TIGAR lowers glycolytic flux and diverts glucose-6-phosphate into the PPP^g^, reducing the dependence on glucose. III. Prior to FX11 treatment, the lactate-to-pyruvate ratio was increased in wild-type TP53 PDAC; following FX11 treatment, the lactate-to-pyruvate ratio decreased in mutant TP53 PDAC	I. HP-MR can be used to confirm the desired effect of metabolic therapies in tumors in early stages of drug development II. The lactate-to-pyruvate ratio can serve as a biomarker for response to metabolic therapies early in the treatment regimen	Wojtkowiak et al [64]
To determine if treating a PDAC cell line with β-lapachone, a chemotherapeutic agent activated by the enzyme NQ01 (upregulated in PDAC), will lead to the breakdown of energetic metabolic pathways such as glycolysis and the tricarboxylic acid cycle (due to depletion of NAD+ and ATP).	I. In vitro model: MIAPaCa2 (NQO1+) pancreatic cancer cells (sensitive to β-lapachone) II. Treatment groups: (i) β-lapachone, (ii) no treatment	[1-^13^C] Pyruvate Pyruvate to lactate	HP [1-^13^C] pyruvate conversion to lactate was lower in cells treated with β-lapachone, suggesting that the activity of LDH is compromised from treatment	HP-MR can noninvasively detect the metabolic response of β-lapachone-treated cells. Thus, it can be used as a direct readout of treatment efficacy in PDAC patients with NQ01 upregulation	Rajeshkumar et al [65]
To determine whether measurement of the apparent diffusion coefficient (ADC) and conversion of injected copolarized ^13^C-labeled pyruvic acid and fumaric acid can detect changes in lactate export and necrosis, respectively	In vitro model: (i) human breast cancer cell line MCF-7 (do not upregulate MCT1 or MCT4 under hypoxic conditions); (ii) mouse PDAC cell line 8932	Mixture of [1-^13^C] pyruvic acid and [1,4-^13^C_2_] fumarate Pyruvate to lactate Fumarate to malate	I. The ADC_lac_-to-ADC_pyr_ ratio is significantly greater in PDAC cells compared to that in MCF-7 cells II. This is corroborated by greater extracellular concentrations from the PDAC line III. Fumarate to malate conversion is detectable only in necrotic cells lysed with Triton X-100; no lactate formation was observed due to dilution of LDH and NADH^h^.	I. Diffusion and conversion of HP pyruvate can provide information about the lactate efflux using the ADC_lac_-to-ADC_pyr_ ratio, which is linked to the relative distribution of lactate in the intra- and extracellular compartments II. Diffusion MR and conversion of HP fumarate can inform necrosis; the rationale is that intracellular ADC<extracellular ADC due to restricted diffusion inside the cell III. Together, the cell’s viability can be assessed. This may be used (1) to localize necrotic areas and (2) to assess the therapeutic response, especially for antiangiogenic agents such as bevacizumab	Silvers et al [54]
To determine whether mice injected with cancer cells (transfected with luciferase) in the peritoneum could be imaged using HP-MR and D_2_O radicals	BALB/cA nu/nu mice: (i) peritoneal metastasis; (ii) intraperitoneal injection of human pancreatic carcinoma (SUIT-2) cells	Free radical (Oxo 63, CmP, nitroxyl)-D_2_O probe	The image intensity correlated positively with the density of malignant ascites in the peritoneum	Radical-D_2_O and HP-MR can be used to selectively visualize H_2_O in the peritoneal cavity of mice and hence detect peritoneal metastasis early; this may then also be used to evaluate drug efficacy	Karlsson et al [66]

^a^GEM: genetically engineered mouse.

^b^PanIN: pancreatic intraepithelial neoplasia.

^c^LDH: lactate dehydrogenase.

^d^ALT: alanine transaminase.

^e^SC: subcutaneous.

^f^PDX: pancreatic ductal adenocarcinoma xenograft.

^g^PPP: pentose phosphate pathway.

^h^NADH: nicotinamide adenine dinucleotide hydrogen.

## Results

### Characteristics of Retrieved Articles

**Figure 3:**
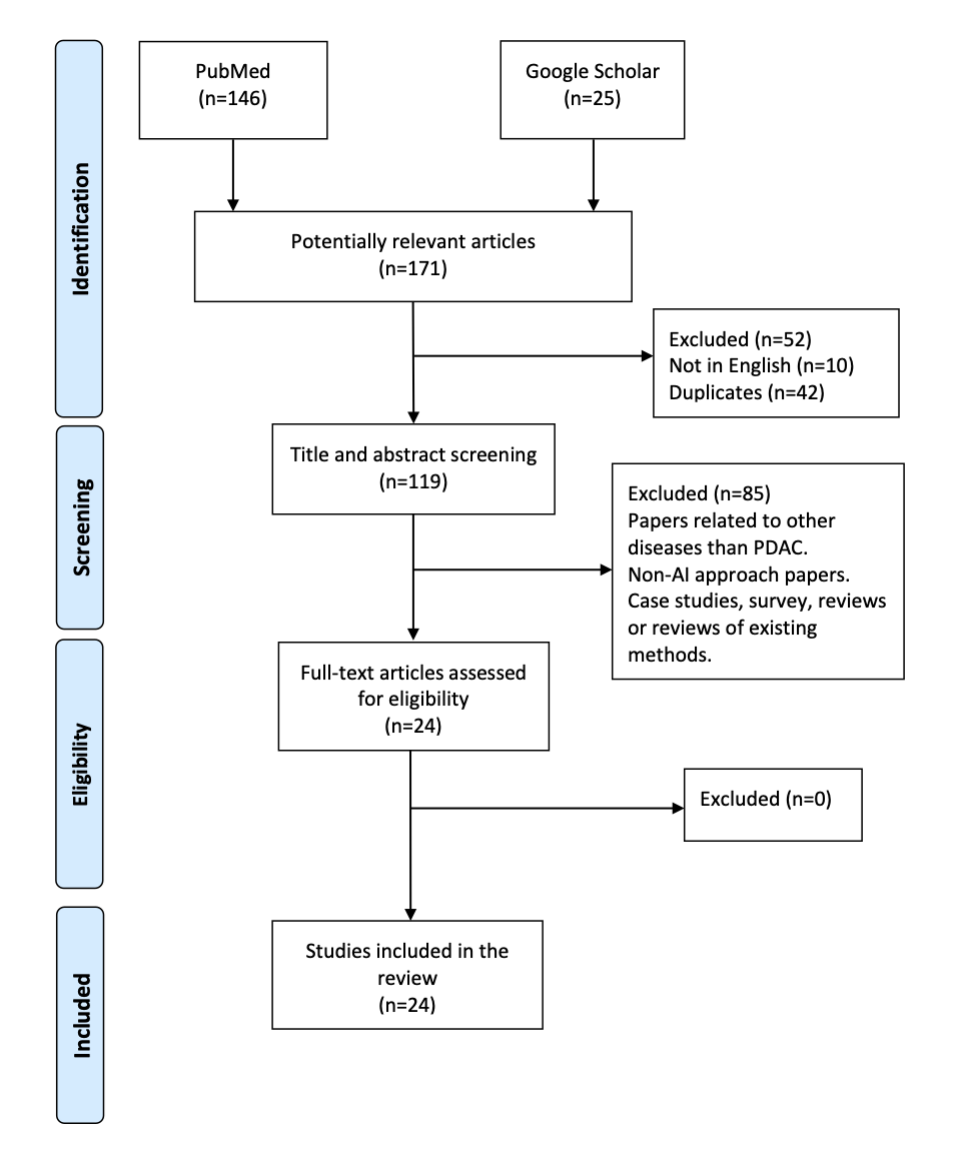
PRISMA flow chart showing the selection criteria of the publications to include in this review. AI: artificial intelligence; PDAC: pancreatic ductal adenocarcinoma.

For AI applications in pancreatic cancer, we retrieved 112 articles from the two sources, including 87 articles from PubMed and 25 articles from Google Scholar. An article was included if it satisfied our inclusion criteria: (1) written in English; (2) utilized AI/machine learning/deep learning for prediction, diagnosis, or classification; and (3) proposed a novel method of employing AI for PDAC (Figure 3). Review, evaluation, and comparison papers were therefore not included. Among the retrieved papers, a total of 17 met the inclusion criteria (Figure 3, Table 2, and Table S1 in Multimedia Appendix 1). The selected papers were grouped into six categories based on how AI was utilized in the context of PDAC to recognize the gaps in the previous studies and to discuss the novel approaches that fill the current gaps in detecting PDAC by imaging modalities.

For HP-MR, we retrieved and reviewed all papers published in this broad area up to November 2020, which included six preclinical studies and one clinical study. We also reviewed several HP-MR–related articles (52 articles) that described new probes that can be applied in many functional future applications in PDAC. These references are not included in the PRISMA flow chart in Figure 3, as they have not yet been demonstrated in PDAC imaging and spectroscopic applications.

### HP Metabolic Imaging Applications in PDAC

#### Context for Application of HP-MR in Pancreatic Cancer

PDAC tumors can be removed by surgery if detected early [23]. There is unequivocal evidence that diagnosis of PDAC at earlier, resectable stages has a profoundly favorable impact on prognosis [1]. The 5-year survival of patients with resected PDAC can reach up to ~25%-30% in major treatment centers, increasing to 30%-60% for tumors <2 cm, and as high as 75% for minute lesions under 10 mm in size [46,47]. Unfortunately, most tumors are diagnosed at a late stage, once advanced into the local blood vessels and other body organs, and can no longer be excised. Thus, there is an urgent call to develop noninvasive imaging modalities for the early detection of PDAC, especially in high-risk patients (eg, those with a familial predisposition, long-standing diabetes, or chronic pancreatitis) [48]. Unlike other cancers such as breast or prostate cancer that have close to 100% survival if detected at early stages, PDAC is associated with a survival rate of only 39% even when detected at stage I [45]. Therefore, there is an urgency to develop novel methods for the detection of preneoplastic lesions in the pancreas.

#### Grading of PDAC

The type of treatment administered is often dependent on the tumor grade; therefore, there is a need for noninvasive methods to determine tumor grade. HP-MR uses metabolic changes to determine a grade [49]. Inside a PDAC tumor, the malignant cells become dependent on glycolysis for energy generation (Warburg effect). Dutta et al [50] recently reported that the aggressiveness of PDAC is directly correlated to pyruvate-to-lactate conversion measured using HP-MR (Table 1) and ex vivo ^1^H nuclear magnetic resonance (NMR) spectroscopy in a panel of well-annotated patient-derived PDAC xenograft (PDX) mouse models. The ex vivo ^1^H NMR spectroscopy results were also in good agreement with in vivo pyruvate-to-lactate conversion, showing a higher abundance of lactate in aggressive tumors. The expression levels of lactate dehydrogenase (LDH)-A and hypoxia-inducing factor-1α were also found to be elevated in aggressive tumors compared to those in less aggressive tumors in PDX mouse tumors. This study demonstrated that the aggressiveness of PDAC could be interrogated noninvasively by employing [1-^13^C] pyruvate with HP-MR [50] to track cellular metabolic activity.

An interesting work by Serrao et al [23] (summarized in Table 2) demonstrated a method for the early detection of PDAC in murine models when the disease is in the early PanIN precursor stage employing HP [1-^13^C] pyruvate. They used genetically engineered mice with K-Ras and p53 mutations or with K-Ras mutation only, which developed PanIN spontaneously. In addition, wild-type mice treated with caerulein injections to induce acute pancreatitis that developed into PanIN over time were included. The mice were imaged using HP [1-^13^C] pyruvate at different stages of development from PanIN to PDAC precursor lesions, and the metabolic fluxes from [1-^13^C] pyruvate to lactate and alanine were measured [23]. The results from individual mice showed a decreasing alanine-to-lactate ratio with disease progression from normal tissue to pancreatitis to low-grade PanIN to high-grade PanIN and finally to PDAC. Mice from all three groups followed this disease progression course, although with disparate timelines. The observed metabolic flux pattern correlated with increasing LDH activity and decreasing alanine transaminase activity. The metabolic flux from pyruvate to lactate and alanine is minimal in normal pancreatic tissue and progressively increases with disease progression. This technique can be used to create 3D metabolic maps of the pancreas to identify the extent of cancerous growth. This work was extended by Dutta et al [62] to demonstrate that real-time conversion kinetic rate constants (k_PA_ and k_PL_) can be used as metabolic imaging biomarkers of premalignant pancreatic lesions. However, the translational potential of this approach can only be ascertained through clinical trials, which is feasible as this emerging technology can be translated to the clinic for the detection of premalignant pancreatic lesions in high-risk populations. Recently, a pilot study reported the feasibility of HP [1-^13^C] pyruvate MRI in PDAC patients, and no adverse effect was observed after bolus injection of pyruvate [63]. These studies reveal the potential for the conversion of HP-pyruvate to lactate in the early detection of PDAC.

In addition, the HP pyruvate-to-lactate ratio may be used for staging tumors in the context of their aggression, although how this paradigm would fit in with the existing standards of staging is debatable (stage I or II: surgically resectable; stage III: locally advanced, unresectable; stage IV: metastatic) [48]. A very promising use of pyruvate-to-lactate flux is to identify PDAC advancing toward stage IV (metastasis) because these tumors show higher pyruvate-to-lactate conversion compared to that of less aggressive pancreatic cancer [50].

#### Early Assessment of Treatment Response

One of the promising utilities of HP-MR is its ability to assess treatment response early during the regimen; this has been established for solid tumors characterized by “aggression correlated with increased glycolysis.” This technique can thus complement the standard fluorodeoxyglucose-positron emission tomography imaging, which can only detect changes in tumor size (rather than intracellular metabolic changes) once it shrinks in response to a long-term regimen of chemotherapy or radiation therapy. Table 2 summarizes four published studies that show how HP-MR can be employed to predict responders (prognostic biomarkers) or assess treatment response early in PDAC tumors or cells [54,63-65]. The treatment efficacy of drugs (hypoxia-activated prodrugs, β-lapachone, and LDH-A inhibitors) evaluated using HP [1-^13^C] pyruvate has only been studied in preclinical models to date; however, the preclinical data illustrate the ability of HP-MR to assist clinical trials by providing a framework for personalized medicine. HP-MR can provide information about the efficacy of drugs at an early stage that can lead to changes in clinical management, enabling the clinician to change the drug for a nonresponding patient to a more effective drug at an early stage.

Wojtkowiak et al [64] (Table 2) screened a hypoxia-activated prodrug (TH-302) as a monotherapy and in combination with pyruvate (not to be confused with the HP probe, [1-^13^C] pyruvate) on three subcutaneous (Hs766t, MIAPaCa-2, and SU.86.86 cells) patient-derived xenografts of PDAC in mice. HP-MR using [1-^13^C] pyruvate was employed to evaluate the metabolic phenotypes of Hs766t, MIAPaCa-2, and SU.86.86 PDAC cell line xenografts. The Hs766t and MIAPaCa-2 xenografts showed higher lactate-to-pyruvate ratios and more hypoxia. However, the SU.86.86 xenograft was resistant to the TH302 hypoxic prodrug because it was less hypoxic. The mice were treated for 2 weeks at a rate of five times a week and tumor sizes were measured at regular intervals with calipers to determine the treatment efficacy. The Hs766t and MIAPaCa-2 groups showed an excellent response with TH302 compared to the SU.86.86 group [64].

Rajeshkumar et al [65] (Table 2) tested the treatment efficacy of the drug FX11, which inhibits LDH-A, on 15 patient-derived PDAC mouse models [65]. LDH-A converts pyruvate to lactate in the presence of its cofactor nicotinamide adenine dinucleotide hydrogen (NADH). Inhibition of LDH-A is a metabolic vulnerability that can be exploited for cancer treatment, and hence FX11 was evaluated in PDAC animal models. The drug was injected once daily for 4 weeks using PDX mouse models with tumors in their flank. The drug efficacy was tested using HP [1-^13^C] pyruvate, which was injected into the mice prior to the start of treatment and 7 days after treatment, prior to any changes in tumor volume. Mice responding to the treatment showed a decreased lactate-to-pyruvate ratio after FX11 administration, whereas nonresponders showed an increased HP lactate-to-pyruvate ratio after the treatment. This result demonstrates the strength of the noninvasive HP-MR modality to predict treatment efficacy prior to tumor size reduction.

The β-lapachone chemotherapeutic drug acts on the quinone oxidoreductase 1 (NQO1)-mediated redox cycle, resulting in elevated superoxide and peroxide formation and in turn nicotinamide adenine dinucleotide (NAD+) depletion due to DNA damage and hyperactivation of poly(ADP-ribose) polymerase. Silvers et al [54] (Table 2) screened β-lapachone on patient-derived MIAPaCa2 cells (which were NQO1+, and hence sensitive to β-lapachone) in vitro to understand the effects of the drug on energy metabolism due to NAD+ depletion. Using metabolic imaging with HP pyruvate, this study showed a decrease in glycolytic flux upon treatment, thus validating the use of HP-MR as a direct readout of the treatment efficacy of β-lapachone in patients with PDAC with upregulated NQO1 expression.

Feuerecker et al [67] (Table 2) took an interesting in vitro approach to understand cancer tumor characteristics such as necrosis and lactate export, which are important parameters to determine cancer aggressiveness. They injected copolarized pyruvate and fumarate to measure the lactate export and necrosis in PDAC and MCF-7 breast carcinoma cells. Increased lactate export and cell necrosis are indicators of tumor aggressiveness, which can be determined using pyruvate-to-lactate flux and fumarate-to-malate flux, respectively. This study measured the apparent diffusion coefficient (ADC) and used HP-MR to examine the necrosis grade. The ADC of intracellular metabolites depends on the intactness of the plasma membrane. A greater ADC_lactate_-to-ADC_pyruvate_ ratio was observed in viable PDAC compared to MCF-7 breast carcinoma cells. The ADC measurements of metabolites could complement the HP lactate-to-pyruvate and HP fumarate-to-malate ratios to determine cell necrosis. This technique can be extended to in vivo measurements to determine the necrotic areas and evaluate the therapeutic response in PDAC patients.

#### Response to Radiation Therapy

Several studies have shown that early responses to radiation therapy can be assessed using molecular imaging. Ionizing radiation generates reactive oxygen species in tumor tissues [68]. Determining oxidative stress noninvasively could measure the extent of oxidative damage. HP pyruvate-to-lactate conversion predicted the response of solid tumors to radiation therapy in animal models [56]. This is an indirect approach and exploits the fact that pyruvate-to-lactate conversion requires reducing equivalents [56]. More direct measurement of redox stress inside cells is provided by HP dehydroascorbate–based MR, as summarized in Table 1 [57-61].

#### Collateral Lethality

Collateral lethality is a novel therapeutic approach that exploits the deletion of passenger genes alongside neighboring (deleted) tumor suppressor genes, thus conferring cancer-specific vulnerabilities [69]. One such instance is the deletion of both copies of malic enzyme 2 (*ME2*) with homozygous deletion of the neighboring *SMAD4* in many cases of PDAC. This makes ME3 inhibition a useful drug target because ME2 and ME3 are paralogous isoforms involved in NADPH regeneration and thus redox balance. The downstream effect of ME3 inhibition entails a reduction in the levels of branched-chain amino acid aminotransferase (BCAT) (encoded by *BCAT2*) via AMP-activated protein kinase–mediated mechanisms [69]. An HP α-keto isocaproate probe (Table 1), which can detect BCAT levels in vivo, could potentially be used for prognosis in the near future [66].

#### Imaging Peritoneal Metastasis

An interesting investigation by Eto et al [70] (Table 2) illustrates a method for the selective imaging of malignant ascites in a mouse model of peritoneal metastasis using HP-MR and bioluminescence studies [70]. In vivo HP images obtained using H_2_O and D_2_O as a radical in SUIT-2 peritoneal metastasis mice showed increasing intensity with time (0, 7, 14, and 21 days after tumor cell administration). This correlated with the increased density of bioluminescence as the density of PDAC ascites increased, thus providing the capability to monitor peritoneal metastasis as well as to evaluate the efficacy of antimetastatic drugs using these two techniques.

#### Metabolic Imaging Employing HP 13C Glutamine

Another possible approach for the early detection of PDAC is using HP ^13^C glutamine. Son et al [51] described a noncanonical metabolic pathway for glutamine observed in PDAC cells. Normal cells convert glutamine-derived glutamate to α-ketoglutarate, which then feeds into the TCA cycle, whereas PDAC cells convert glutamine-derived glutamate into aspartate inside the mitochondria. This aspartate migrates to the cytosol and undergoes further biochemical reactions, which ultimately contribute to redox balance. This study also stated that the pathway is dispensable in normal cells (inhibiting the enzymes of this pathway is easily tolerated by normal cells), but is crucial to the survival of PDAC cells. However, it is not clear whether the pathway of glutamine to aspartate via glutamate is more pronounced in PDAC as compared to the normal tissue. If the glutamine to aspartate via glutamate pathway is upregulated by several fold compared to that in normal cells, this metabolic pathway can be exploited to diagnose and grade PDAC tumors employing HP-MR with HP [^13^C] glutamine. The feasibility of this approach depends on several factors. First, the decay time for HP ^13^C glutamine must be considerably longer than the uptake of glutamine by PDAC cells, and the time of conversion to glutamate and then to aspartate. Additionally, there needs to be preferential uptake in PDAC cells compared to the cells of the normal pancreas. HP glutamine has already been used to study cancer cells from other tumor types [71] (Table 1).

#### Interstitial pH Mapping

Pancreatitis (inflammation of the pancreas) and PDAC are characterized by acidic microenvironments. The interstitial pH of the pancreas is reduced in patients with chronic pancreatitis [72-74]. The use of pH imaging to differentiate the acidic microenvironment of pancreatic tumors from that of PanIN lesions in mice has been elucidated by Cruz-Monserrate et al [52]. Several HP probes such as bicarbonate and zymonic acid can be potentially employed to image extracellular pH in tissue, which are summarized in Table 1 [22,53,55,67,75].

### AI Applications in PDAC

#### Overview of AI and Deep Learning for PDAC

Deep learning has shown robust and extraordinary performance in medical image analysis. Many previous studies have explored the applications of AI, especially deep learning, for diagnosing and detecting various diseases, including pancreatic cancer, from different imaging modalities [76,77]. Leveraging HP-MR with deep learning is a promising approach to interrogate the early diagnosis and early efficacy of therapy for pancreatic cancer.

Most of the innovative applications of deep learning in biomedical imaging were triggered by convolutional neural networks (CNNs) [78], a powerful method for representation learning in images and structured data. As discussed above, neural networks, inspired by information transformation in the biological brain, require connections of all nodes of one layer to the next, which is insufficient for image analysis and fails to make use of spatial information. To overcome these issues, CNN introduces convolutional layers and pooling operations. In addition, many innovative modifications have been proposed to boost the performance of CNN, including dropout [79], batch normalization [80], and residual learning [81]. Essentially, the input to CNN is in a grid structure to preserve the spatial information, and then multiple convolutional layers and activation layers, interspersed with pooling layers, are utilized to process the data and learn structure in each level. Furthermore, a fully connected layer computes the final outputs for image analysis tasks.

A convolutional layer includes a set of filters with learnable parameters. Each filter is slid across the width and height of the input, and the dot product of the filter and input at every special position is calculated and goes through an activation function. A nonlinear activation function, typically rectified linear units (ReLUs), expands the potential in approximation of any nonlinear function [82]. The output of a convolution layer is a stack of activation maps of all filters. For pooling layers, it takes small regions in the feature map and produces a single number as the output to extract the most significant information learned from convolutional layers.

Several variants of CNNs with innovative architectures have been proposed to achieve better performance on specific tasks or types of data. VGG [83] introduced smaller filter kernels and constructed a deeper network compared with AlexNet [84], which first utilized ReLUs, dropout, and GPU accelerations. ResNet [81] proposed residual learning by using skip connections, which not only reduces the number of parameters but also makes the network deeper at up to 152 layers without a vanishing gradient. For biomedical images, U-Net [85] constructed downstreaming and upstreaming paths for biomedical images processing, connected by a skip connection, which concatenates features to the upstreaming path. V-Net [86] extended U-Net to 3D datasets using 3D convolutional layers and achieved extraordinary performance.

To review the previous studies on using AI for PDAC, we grouped the 17 selected papers (Table 3 and Table S1 in Multimedia Appendix 1) meeting our inclusion criteria into six categories based on how AI was utilized in the context of PDAC to help recognize the gaps in the previous studies and to discuss the novel approaches that can fill the current gaps in detecting PDAC by imaging modalities.

**Table 3:** Review of published applications of artificial intelligence for pancreatic ductal adenocarcinoma (PDAC).

Reference	Task	Method	Dataset	Performance
Liu et al [87]	A patient-specific tumor growth model based on longitudinal multimodal imaging data, including dual-phase CT^a^ and FDG-PET^b^	A coupled PDE^c^ system to develop a reaction-diffusion model enabling the incorporation of the cell metabolic rate and calculate ICVF^d^	Average ICVF difference (AICVFD) of tumor surface and tumor relative volume difference (RVD) on six patients with pathologically confirmed pancreatic neuroendocrine tumors	The ASD^e^ between the predicted tumor and the reference tumor was 2.4 mm (SD 0.5), the RMSD^f^ was 4.3% (SD 0.4), the AICVFD was 2.6% (SD 0.6), and the RVD was 7.7% (SD 1.3)
Fu et al [88]	CT pancreas segmentation (edge detection)	Proposed model includes 13 convolutional layers and 4 pooling layers; introduced multilayer upsampling structure	CT images from the General Surgery Department of Peking Union Medical College Hospital; 59 patients, including 15 with nonpancreas diseases and 44 with pancreas-related diseases	76.36% DSC^g^
Gibson et al [89]	Multiorgan segmentation on abdominal CT	Modified V-net proposed by replacing the convolutional layers in the encoder path by DenseNet consisting of stacks of dense blocks combined with bilinear upsampling in the decoder path	Two publicly available datasets: 43 subjects from the Cancer Imaging Archive Pancreas CT dataset with pancreas segmentations and 47 subjects from the Beyond the Cranial Vault segmentation challenge with segmentations of all organs except the duodenum	DSC of 78% for the pancreas, 90% for the stomach, and 76% for the esophagus
Luo et al [90]	Preoperative prediction of pancreatic neuroendocrine neoplasms (pNENs) grading by CECT^h^	The proposed 3D CNN^i^ composed of 1 CNN layer with 1 rectifier linear unit layer, a max pooling layer, 12 IdentityBlock, 4 ConvBlock, 1 global average pooling layer, and 1 fully connected layer	CT images of 93 patients from Sun Yat-Sen University and 19 patients from The Cancer Center of Sun Yat-Sen University with pathologically confirmed pNENs	AUC^j^ of 0.81
Liu et al [91]	Diagnosis of pancreatic cancer using CNN	Pretrained VGG16 serves as a feature extraction network, and Faster R-CNN is used for diagnosis	6084 enhanced CT horizontal images from 338 pancreatic cancer patients	AUC of 0.96
Boers et al [92]	Segmentation of the pancreas	U-net model was changed by adding one interactive layer that takes feedback from the annotator while freezing other layers to do retraining	Public dataset (Gibson et al [89]), which contains 90 late venous-phase abdominal CT images	DSC of 78.1% (SD 8.7)
Liu et al [93]	Cone-beam CT (CBCT) quality and HU^k^ accuracy improvement	A self-attention cycle generative adversarial network (cycleGAN) was used to generate CBCT from synthetic CT	Thirty patients previously treated with pancreas SBRT^l^ at Emory University	Mean absolute error between CT and synthetic CT of 56.89 (SD 13.84) HU and 1.06 (SD 15.86) HU between CT and the raw CBCT
Park et al [94]	CT data collection for deep learning	Two U-Net models were linked by an organ-attention module	From 575 participants, a total of 1150 CT images	Mean DSC of 89.4% and mean surface distance of 1.29 mm
Liu et al [95]	Multiorgan segmentation for pancreatic CT	3D U‐Net with an attention strategy is proposed	100 patients with CT simulation scanned	DSC of 91% (SD 3), 89% (SD 6), 86% (SD 6), 95% (SD 2), 95% (SD 2), 96% (SD 1), 87% (SD 5), and 93% (SD 3) for the large bowel, small bowel, duodenum, left kidney, right kidney, liver, spinal cord, and stomach, respectively.
Mu et al [96]	Prediction of clinically relevant postoperative pancreatic fistula using CECT	One convblock, 8 residual blocks, and one fully connected layer	A group of 513 patients underwent pancreaticoenteric anastomosis after PD^m^ at three institutions between 2006 and 2019	AUC of 0.89
Chu et al [77]	Deep-learning models for abdominal organs segmentation using CT	Three networks with different voxel sizes. Each network follows an encoder-decoder topology and includes a series of CNN layers max pooling and deconvolutional layers	Dual-phase CT from 575 control subjects and 750 patients with PDAC from 2005 to 2017	Accuracy of 87.8% (SD 3.1)
Suman et al [97]	Deep-learning models for pancreas segmentation using CT	NVIDIA 3D Slicer segmentation module	347 CECT scans based on a statement of a negative or unremarkable pancreas in the original radiologist’s report	DSC of 63% (SD 15)
Ma et al [98]	Pancreatic cancer diagnosis using CT	The model consisted of three convolutional layers and a fully connected layer	3494 CT images from 222 patients with pathologically confirmed pancreatic cancer and 3751 CT images from 190 patients with normal pancreas from June 2017 to June 2018	Accuracy of 82.06%, 79.06%, and 78.80% on plain phase, arterial phase, and venous phase
Zhang et al [99]	Tumor detection framework for pancreatic cancer via CECT	Feature pyramid networks with Faster R-CNN	2890 CT images from Qingdao University	AUC of 0.9455
Corral et al [100]	Intraductal papillary mucinous neoplasms (IPMN) classification using MRI^n^	Integration of CNN and SVM^o^	171 patients, 39 MRIs with no pancreatic lesions served, and 132 confirmed IPMN	AUC of 0.77
Hussein et al [101]	IPMN classification using MRI	VGG network and SVM	171 MRIs for 38 subjects with normal pancreas, and the remaining 133 from subjects diagnosed with IPMN	Accuracy of 84.22%
Liang et al [102]	MRI pancreas segmentation	SVM with recursively retraining samples	MRIs from four patients with locally advanced pancreatic cancer	DSC of 86%
Zheng et al [103]	MRI pancreas segmentation	2D Unet	20 patients with PDAC	DSC of 73.88%

^a^CT: computed tomography.

^b^FDG-PET: fluorodeoxyglucose-positron emission tomography.

^c^PDE: partial differential equation.

^d^ICVF: intracellular volume fraction.

^e^ASD: average surface distance.

^f^RMSD: root mean square deviation.

^g^DSC: Dice similarity coefficient.

^h^CECT: contrast-enhanced computed tomography.

^i^CNN: convolutional neural network.

^j^AUC: area under the receiver operating characteristic curve.

^k^HU: Hounsfield unit.

^l^SBRT: stereotactic body radiotherapy.

^m^PD: pancreatoduodenectomy.

^n^MRI: magnetic resonance imaging.

^o^SVM: support vector machine.

#### Tumor Growth Model

Tumor growth, especially for pancreatic neuroendocrine tumors, is related to cancer cell properties and relies on the dynamic interaction between cells and the microenvironment. Swanson et al [104] proposed a reaction-diffusion model by assuming infiltrative growth of the tumor cells but did not consider the cell metabolic rate. Liu et al [87] introduced dual-phase CT-measured intracellular volume fraction (ICVF) to the reaction-diffusion model. Cell metabolic rate was considered in the prediction of pancreatic neuroendocrine tumor growth. They evaluated the model by comparing predictions with sequential observations regarding average surface distance, root mean square deviation (RMSD) of the ICVF map, and average ICVF difference in six patients with pancreatic neuroendocrine tumors. Although the RMSD was around 4.3%, the limited number of patients involved might have undermined the final findings.

#### Organ/Multiorgan Segmentation and Edge Detection in Medical Images

Fu et al [88] discussed the application of a CNN consisting of 13 convolution layers and 4 pooling layers with a multilayer upsampling structure in pancreas segmentation from CT images. The proposed model was evaluated using real PDAC CT images from a dataset created by the General Surgery Department of Peking Union Medical College Hospital. The 59 patients consisted of 15 patients with nonpancreas diseases and 44 patients with pancreas-related diseases. A Dice similarity coefficient (DSC) of 76.36% was achieved. The introduced fusion layer provided good visualization for decision-making and multilayer upsampling improved the performance. However, due to the limited number of CT images for training and validation, its performance suffered from the risk of overfitting as the reported SD from precision 5-fold cross-validation was very high (mean SD of 18.08 across all classes). Moreover, the reported precision and recall for the healthy cohort (80.95 and 86.53, respectively) was much higher than that for the IPMN (75.39 and 67.37) or pancreatic neuroendocrine tumor (70.44 and 74.86) cohort.

Alternatively, to implement multiorgan segmentation, especially on abdominal CT in the pancreas, Gibson et al [89] modified V-net by replacing the convolutional layers in the encoder path by DenseNet consisting of stacks of dense blocks combined with bilinear upsampling in the decoder path. They applied this on two public datasets: one including 43 subjects from the Cancer Imaging Archive Pancreas CT data with pancreas segmentation, and the other including 47 subjects from the Beyond the Cranial Vault segmentation challenge with segmentations of all organs except the duodenum. They achieved a DSC of 78%. The introduced dense feature stack considerably reduced the number of parameters for medical image classification tasks. However, this approach is only appropriate for relatively small datasets because of overfitting issues.

As another example of an attempt to improve pancreas segmentation performance in CT scans, Boers et al [92] developed an interactive version of U-net (iUnet) by adding one interactive layer after the last fully connected layer takes feedback from annotators while freezing other layers to do the retraining. This was applied to a public CT dataset used in Gibson et al [89], which contains 90 late venous-phase abdominal CT images and a respective reference segmentation. A DSC of 78.1% (SD 8.7%) was achieved from the interactive version of iUnet, which outperformed previous methods using the same dataset. However, this approach may also suffer from overfitting issues since interactive processes may introduce external information, which limits its scalability.

Liu et al [93] presented a deep-attention U-net approach to solve multiorgan segmentation for pancreatic cancer CT images. This method achieved state-of-the-art performance, but its performance in pancreas segmentation is unclear.

Besides CT images, investigators using T1-MRI proposed several innovative approaches to segment the pancreas. Liang et al [102] introduced a top-down and bottom-up approach. In the top-down path, the initial planning contours derived from simulation MR images are transferred to daily images, and in the bottom-up path, the probabilistic support vector machine (SVM) is used with recursively retraining samples. The final result is obtained by fusing both paths and the final reported DSC was 86%. Zheng et al [103] proposed a 2D U-Net approach with shadow sets for MRI and CT pancreas segmentation. The usage of shadow sets reduced uncertainty and achieved a DSC of 84.37% on the NIH-CT-82 public dataset [105] and 73.88% on an MRI dataset collected from Changhai Hospital, including 20 patients with PDAC.

#### Prediction of PDAC and Risk Evaluation

To implement preoperative prediction of pancreatic neuroendocrine neoplasms (pNENs) grading by CT, Luo et al [90] applied a CNN model with identity blocks and convolution blocks to a CT imaging dataset consisting of 93 patients from the hospital. An arterial model employed for the pathological grading of pNENs achieved an area under the receiver operating characteristic curve (AUC) of 0.81. Due to the limitations of the dataset, simple deep-learning models may undermine the feature extraction ability and lead to suboptimal performance. In addition, the limited observations may lead to a lack of an independent evaluation dataset and invalidation of n-fold cross-validation, which constrains the scalability and generalizability of the proposed model.

For the prediction of clinically relevant postoperative pancreatic fistula using CT images, Mu et al [96] utilized a Resnet18 model with fewer filters on 513 patients imaged between 2006 and 2019. All patients underwent pancreatico-enteric anastomosis after the diagnosis of pancreas disease at three institutions. Compared to the commonly used Fistula Risk Score (FRS), the proposed model improved the prediction AUC from 0.73 to 0.89. This study illustrated that deep learning might overcome intermediate risk score issues in FRS with greater predictability.

Hussein et al [101] utilized clustering and SVM with initial label estimation for risk stratification of pancreatic tumors. The model outperformed other unsupervised methods, achieving 58% accuracy out of 171 scans, of which 38 subjects were normal and the remaining 133 were diagnosed with IPMNs. In addition, Corral et al [100] performed similar experiments using MRI with CNN and SVM as the final classifier, and achieved sensitivity and specificity of 0.92 and 0.52, respectively, for the detection of dysplasia. In this task, the deep-learning protocol barely outperformed radiologists due to unbalanced data issues and the complexity of IPMN.

#### Diagnosis of PDAC

To implement diagnosis of PDAC from CT images, Liu et al [91] utilized a pretrained VGG16 model as a feature extraction network in conjunction with a faster recurrent CNN (R-CNN) as a decision-maker. Their CT dataset consisted of 6084 enhanced CT horizontal images from the abdomen of 338 PDAC patients. This achieved an AUC of 0.9632 for the prediction of PDAC. R-CNN usage for sequential information extraction greatly improved the diagnostic performance, and its dynamic feature extraction provided model interpretability and scalability.

Ma et al [98] utilized a regular CNN with four hidden layers on 3494 CT images from 222 patients with pathologically confirmed PDAC and 3751 CT images from 190 patients with a normal pancreas as controls from the First Affiliated Hospital, Zhejiang University School of Medicine. The overall diagnostic accuracy of trained binary classifiers was over 95%. However, it failed to beat human performance. With a similar data size, Liu et al [91] achieved better performance using a more complicated, faster R-CNN, implying the complexity of pancreatic cancer detection and its need for an appropriate model architecture design and parameter fine-tuning.

Zhang et al [99] proposed a tumor detection framework for PDAC using CT. The framework utilized feature pyramid networks with the faster R-CNN model. The Affiliated Hospital of Qingdao University provided a dataset containing 2890 CT images, and a classification AUC of 0.9455 was achieved. This framework outperformed the state-of-the-art methods, but still suffered from input uncertainty inherent in closed-source datasets. The comparison would be more effective if a public dataset was used in the experiment.

#### Improvement of Image Quality

Stereotactic body radiotherapy (SBRT) has shown more success in patients with locally advanced pancreatic cancer compared to conventional radiotherapy. To overcome interference due to motion in the breathing cycle and patient weight loss [106], cone-beam CT (CBCT) is commonly used for target position verification and setup displacement correction to avoid suboptimal target coverage and excessive doses to organs at risk. However, raw CBCT data cannot be used for SBRT dosage calculation due to considerable artifacts such as streaking and shading [107-109] caused by scatter contamination, resulting in different Hounsfield unit (HU) values from CT scans [110]. To improve the HU fidelity of CBCT, Liu et al [95] utilized self-attention cycleGan-based CBCT to synthetic CT (sCT) models on a dataset consisting of 30 patients previously treated with pancreas SBRT at Emory University. The mean absolute error of the proposed framework between CT and sCT was 56.89 (SD 13.84) HU compared to 81.06 (SD 15.86) HU between CT and the raw CBCT.

#### Criteria to Evaluate Annotation Accuracy in Medical Images

To test the CT data collection quality, Park et al [94] proposed two U-net–linked networks, linked by an organ-attention module, to test the performance of a well-annotated dataset, including a total of 575 participants and 1150 CT images. After appropriate management of the annotation process, an average DSC of 89.4% was achieved. This study innovatively employed a deep-learning model to test CT image annotation performance, and improved the annotation quality for further analysis and research. However, this approach still suffers from uncertainty introduced by model training and simulation.

Suman et al [97] used CNNs to train technologists in labeling pancreas segmentation CT datasets. DSC was improved through interactions between model output and expert correction, which implied that annotation quality was enhanced.

## Discussion

In this review, we have discussed how two different techniques, HP-MR and AI, are revealing exciting information about PDAC and PanINs that was not accessible by diagnostic imaging even a few years ago. Deep-learning models eliminate the requirement of domain knowledge for feature engineering that is necessary for conventional machine learning models by learning from raw data. Deep-learning models are capable of learning features from the raw data and apply nonlinear transformations to map the input data to high-dimensional representations trivializing classification or regression. These models are uniquely able to transform multiple modalities into common latent space to synthesize features across all modalities to improve classification performance. However, there is no free lunch, and the flexibility and high accuracy resulting from millions of parameters comes with a requirement of a huge training dataset in comparison with other machine-learning techniques. Moreover, these models suffer from lack of interpretability and uncertainty measurement. In machine-learning algorithms, there is a tradeoff between interpretability and accuracy. When the prediction accuracy grows with more complex (increase in the number of trainable parameters) deep-learning models, the interpretability decreases. For instance, ResNet contains 5×10^7^ parameters requiring 10^10^ floating point operations for a single classification task, making it almost impossible to be traced or explained by humans [81,111]. Lastly, deep-learning models do not provide any uncertainty measurement to measure how certain the model is with its prediction. These models are blindly used with the assumption of “good accuracy,” whereas previous experience has shown that these models are susceptible to overconfident decision-making, especially when the new data are far from the training data distribution (corner case). The lack of interpretability and uncertainty estimation is even more serious in clinical decision-making tasks since it is needed for building trust in the model’s prediction.

Studies on HP-MR have demonstrated that this modality can detect metabolic changes at very early stages of lesion formation in the pancreas (eg, PanIN 1 and 2); however, this is more of a spectroscopic technique than an imaging modality. Furthermore, the signal from HP compounds lasts no more than a few minutes depending on the T_1_ that allows for rapid acquisition of dynamic metabolic flux in the organ of interest. Table 4 summarizes the strengths and weaknesses of AI, MRI, and HP-MR.

**Table 4:** Strengths and weaknesses of artificial intelligence (AI), magnetic resonance imaging (MRI), and hyperpolarized magnetic resonance (HP-MR).

Technique	Strengths	Weaknesses
MRI	Rapid acquisition of anatomical images. Well-established and widely distributed imaging modality.	Poor signal-to-noise ratio and contrast-to-noise ratio. Cannot detect pancreatic cancer at early stages.
HP-MR	Real-time metabolic flux measurements at the organ of interest. Can detect premalignant stages of pancreatic cancer.	Short time window of imaging (~2 minutes). Expensive initial investment in the infrastructure. Slow adoption in the clinical setting.
AI	No feature engineering, ability to learn features from raw data. Ability to learn features from and across multiple modalities. High accuracy result.	Intensive data requirement. High uncertainty on corner cases. Lack of interpretability.

**Figure 4:**
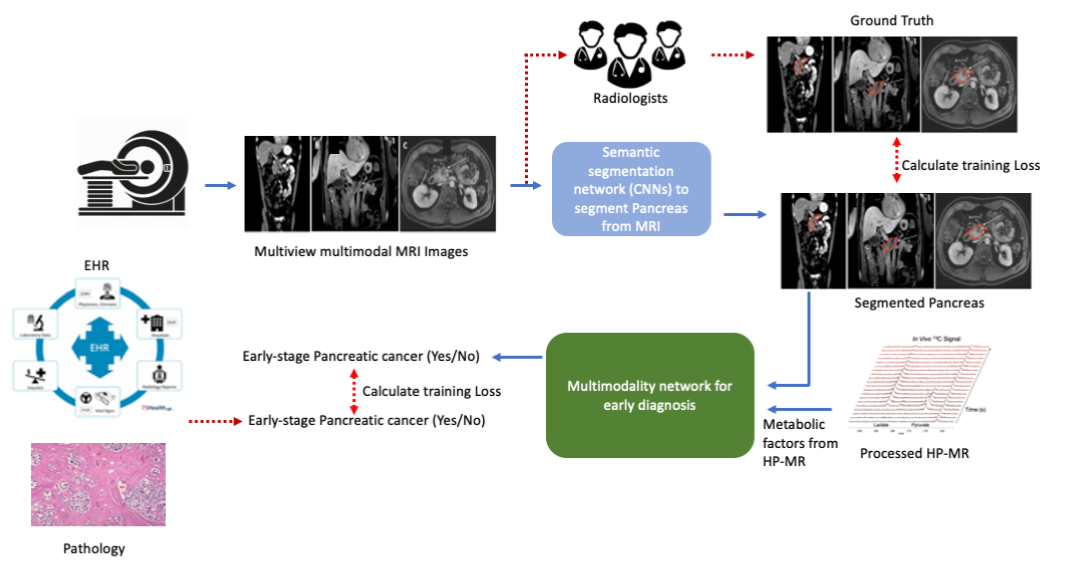
Schematic illustrating the concept of leveraging anatomical magnetic resonance imaging (MRI), hyperpolarized magnetic resonance (HP-MR), and artificial intelligence as complementary modalities toward developing actionable biomarkers of pancreatic ductal adenocarcinoma. CNNs: convolutional neural networks; EHR: electronic health record.

To take advantage of the strengths of AI, MRI, and HP-MR, and mitigate their weaknesses, we propose the following pipeline as illustrated in Figure 4. Our pipeline leverages the unique capability of AI to learn features from each and across both HP-MR and MRI as complementary modalities to investigate the early detection of PDAC by overlaying the anatomical imaging for localized spectroscopic information of real-time metabolic flux in the pancreas. Additionally, we utilize Grad-CAM [112,113] and concrete dropout to provide a visual explanation, and introduce Bayesian inference to estimate uncertainty in the model’s decision.

The training process of our pipeline is as follows: axial, sagittal, and coronal MR images in the T1 and T2 modalities are annotated to highlight the pancreas area by radiologists to train a deep-learning semantic segmentation network developed by our team. We extract the ROIs from MR images (ie, the pancreas). The extracted ROIs with metabolic information from HP-MR are the inputs for our multimodal deep-learning model to predict pancreatic cancer status. The appropriate combination of MRI and HP-MR as complementary modalities improves the classification performance. Therefore, the ground truth for our second deep-learning model is the presence of early stages of PDAC established by pathology reports and electronic health records of the patients. The training path is shown with the dashed lines and the inference path is shown with the solid lines in Figure 4. It has been estimated that there is a window of opportunity of ~10 years from the moment in which a pancreatic epithelial cell undergoes an oncogenic hit and the time of diagnosis of, often fatal, pancreatic cancer [46,114]. Together, AI, HP-MR, and conventional MRI as complementary modalities can address this knowledge gap in diagnostic imaging within this crucial time window of opportunity to save lives.

Leveraging AI and HP-MR applications together may lead to the development of real-time actionable biomarkers of early detection, assessing aggressiveness, and interrogating the early efficacy of therapy in PDAC. For example, multimodal AI can learn features from both HP-MR, as well as anatomical MRI and CT imaging modalities, to yield “hybrid biomarkers” and reduce the time required to detect PDAC evolution in three key areas of tumor progression: initial development of the tumor, its regression following therapy, and the eventual recurrence of the tumor. This innovative synthesis of these techniques may result in a more sensitive readout of tumor progression that can be readily translated and significantly impact how PDAC patients, as well as patients at high risk of developing this deadly disease, are currently managed in the clinic.

## Appendix

Multimedia Appendix 1 Supplemental material.

## Abbreviations

ADC: apparent diffusion coefficient
AI: artificial intelligence
AUC: area under the receiver operating characteristic curve
BCAT: branched-chain amino acid aminotransferase
CBCT: cone-beam computed tomography
CNN: convolutional neural network
CT: computed tomography
DNP: dynamic nuclear polarization
DSC: Dice similarity coefficient
FRS: Fistula Risk Score
HP: hyperpolarized
HU: Hounsfield unit
ICVF: intracellular volume fraction
IPMN: intraductal papillary mucinous neoplasms
LDH: lactate dehydrogenase
MR: magnetic resonance
MRI: magnetic resonance imaging
NAD+: nicotinamide adenine dinucleotide
NADH: nicotinamide adenine dinucleotide hydrogen
NQO1: quinone oxidoreductase 1
PanIN: pancreatic intraepithelial neoplasia
PDAC: pancreatic ductal adenocarcinoma
PDX: patient-derived xenograft
pNEN: pancreatic neuroendocrine neoplasm
PRISMA-ScR: Preferred Reporting Items for Systematic Reviews and Meta-analysis Extension for Scoping Reviews
R-CNN: recurrent convolutional neural network
ReLU: rectified linear unit
ROI: region of interest
SBRT: stereotactic body radiation therapy
sCT: synthetic computed tomography
SVM: support vector machine
T1: longitudinal relaxation time
TCA: tricarboxylic acid
