# Supplementary material for: Hyperpolarized Magnetic Resonance and Artificial Intelligence: Frontiers of Imaging in Pancreatic Cancer
Source: JMIR Med Inform. 2021 Jun 17;9(6):e26601. doi: 10.2196/26601 (PMC8277399; doi:10.2196/26601)
Supplement: Multimedia Appendix 1 [file medinform_v9i6e26601_app1.docx]

## Supplemental Material


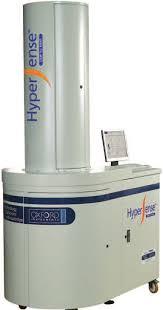

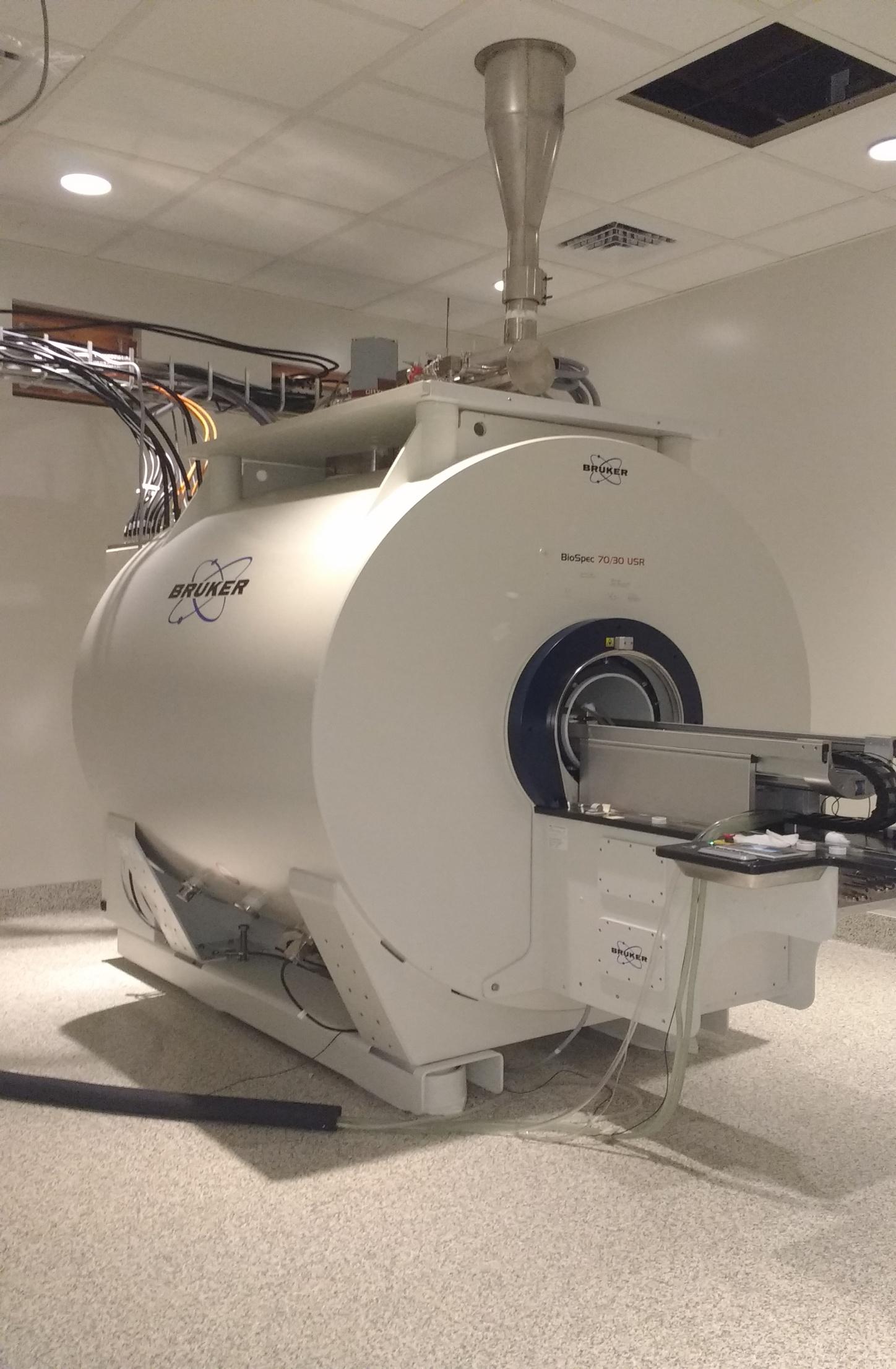


**A**

**B**

**Figure S1** **A**: A photo of a HyperSense DNP system. The cabinet at the bottom is engineered to achieve temperatures near 1K, where a ^13^C sample doped with free radical is irradiated with microwaves to achieve solid-state hyperpolarization. The upper cabinet is used for sample insertion and retrieval, with the help of a long tube that is fitted with a sample cup. **B**: A preclinical Bruker 7T MRI scanner. Due to the short decay time of HP pyruvate probe (less than 120s), the HyperSense and MRI scanner must be in proximity, to allow instant injection of probe following hyperpolarization.

**Table S1:** Summary of the papers on AI applications in PDAC included in this review.

| Title | Year | Journal | Citation | | Keywords |
| --- | --- | --- | --- | --- | --- |
| Patient specific tumor growth prediction using multimodal images. [88] | 2014 | Medical Image Analysis | 58 | | Tumor growth modeling; Multimodal images; Intracellular Volume Fraction; Metabolic rate |
| Hierarchical combinatorial deep learning architecture for pancreas segmentation of medical computed tomography cancer images. [89] | 2018 | BMC Systems Biology | 19 | | Multi-layer up-sampling structure; Pancreas segmentation; Single object segmentation |
| Automatic Multi-Organ Segmentation on Abdominal CT With Dense V-Networks. [90] | 2018 | IEEE Transactions on Medical Imaging | 176 | | Abdominal CT; Segmentation; Deep learning; Pancreas; Gastrointestinal tract; Stomach; Duodenum; Esophagus; Liver; Spleen; Kidney; Gallbladder |
| Preoperative Prediction of Pancreatic Neuroendocrine Neoplasms Grading Based on Enhanced Computed Tomography Imaging: Validation of Deep Learning with a Convolutional Neural Network. [96] | 2020 | Neuroendocrinology | 4 | | Pancreatic neoplasms · Neuroendocrine tumors · Deep learning · Tomography; spiral computed · Neoplasm grading |
| Establishment and application of an artificial intelligence diagnosis system for pancreatic cancer with a faster region-based Convolutional Neural Network. [100] | 2019 | Chinese Medical Journal | 4 | | Artificial intelligence; Pancreatic cancer; Diagnosis; Faster region-based Convolutional Neural Network |
| Interactive 3D U-net for the segmentation of the pancreas in computed tomography scans. [91] | 2020 | Physics in Medicine and Biology | 2 | | Deep learning; Pancreatic cancer; Interactive segmentation; U-net |
| CBCT-based synthetic CT generation using deep-attention cycle-GAN for pancreatic adaptive radiotherapy. [108] | 2020 | Medical Physics | 7 | | Self‐attention cycle; GANCBCT‐based synthetic CT generation; adaptive radiotherapy |
| Annotated normal CT data of the abdomen for deep learning: Challenges and strategies for implementation. [109] | 2020 | Diagnostic and Interventional Imaging | 11 | | Abdominal computed tomography (CT); Image segmentation; Machine learning; Normal structures; Artificial intelligence (AI) |
| CT-based multi-organ segmentation using a 3D self-attention U-net network for pancreatic radiotherapy. [92] | 2020 | Medical Physics | NA | | Adaptive radiotherapy; Multi‐organ segmentation; Pancreatic radiotherapy; Treatment planning |
| Prediction of clinically relevant Pancreatico-enteric Anastomotic Fistulas after Pancreatoduodenectomy using deep learning of Preoperative Computed Tomography. [97] | 2020 | Theranostics | NA | | Pancreatic fistula; Fistula risk score; Pancreatoduodenectomy; Computed Tomography (CT); Deep learning |
| Application of Deep Learning to Pancreatic Cancer Detection: Lessons Learned From Our Initial Experience. [77] | 2019 | Journal of American College of Radiology | 16 | | NA |
| Development of a volumetric pancreas segmentation CT dataset for AI applications through trained technologists: a study during the COVID 19 containment phase. [110] | 2020 | Abdominal Radiology | | NA | Deep learning; Data curation; Artificial intelligence; COVID-19 |
| Construction of a Convolutional Neural Network classifier developed by computed tomography images for pancreatic cancer diagnosis. [101] | 2020 | World Journal of Gastroenterology | | NA | Deep learning; Convolutional Neural Networks; Pancreatic cancer; Computed tomography |
| A Novel and Efficient Tumor Detection Framework for Pancreatic Cancer via CT Images. [102] | 2020 | Annual International Conference of the IEEE Engineering in Medicine & Biology Society (EMBC) | | NA | Tumors; Feature extraction; Computed tomography; Cancer; Proposals; Training |
| Deep Learning to Classify Intraductal Papillary Mucinous Neoplasms Using Magnetic Resonance Imaging. [99] | 2019 | Pancreas | | 7 | Abdominal imaging; Deep learning; Intraductal papillary mucinous neoplasm; Magnetic resonance imaging |
| Lung and Pancreatic Tumor Characterization in the Deep Learning Era: Novel Supervised and Unsupervised Learning Approaches. [98] | 2019 | IEEE Transactions on Medical Imaging | | 39 | Lung; Unsupervised learning; Tumors, Cancer; Three-dimensional displays; Feature extraction; Deep learning |
| Abdominal, multi-organ, auto-contouring method for online adaptive magnetic resonance guided radiotherapy: An intelligent, multi-level fusion approach. [93] | 2018 | Artificial Intelligence in Medicine | | 17 | Auto-Contouring; Machine learning; Adaptive radiotherapy; Image-guided Radiotherapy |
| Deep pancreas segmentation with uncertain regions of shadowed sets. [94] | 2020 | Magnetic Resonance Imaging | | 1 | Pancreas segmentation; Shadowed sets; Uncertainty |
